# Antitumor Effects of Natural Compounds Derived from *Allium sativum* on Neuroblastoma: An Overview

**DOI:** 10.3390/antiox11010048

**Published:** 2021-12-27

**Authors:** Carlos César Patiño-Morales, Ricardo Jaime-Cruz, Concepción Sánchez-Gómez, Juan Carlos Corona, Estefani Yaquelin Hernández-Cruz, Ivia Kalinova-Jelezova, José Pedraza-Chaverri, Perla D. Maldonado, Carlos Alfredo Silva-Islas, Marcela Salazar-García

**Affiliations:** 1 Laboratory of Cell Biology, Universidad Autónoma Metropolitana—Cuajimalpa, Mexico City 05348, Mexico; cpatino@cua.uam.mx; 2 Laboratory of Developmental Biology and Experimental Teratogenesis, Hospital Infantil de México Federico Gómez, Mexico City 06720, Mexico; rjc@xanum.uam.mx (R.J.-C.); csanchez@himfg.edu.mx (C.S.-G.); 3 Laboratory of Neurosciences, Hospital Infantil de México Federico Gómez, Mexico City 06720, Mexico; jcorona@himfg.edu.mx; 4 Department of Biology, Faculty of Chemistry, Universidad Nacional Autónoma de México, Mexico City 04510, Mexico; estefani.hernandez@quimica.unam.mx (E.Y.H.-C.); iviakj@comunidad.unam.mx (I.K.-J.); pedraza@unam.mx (J.P.-C.); 5 Laboratory of Cerebral Vascular Pathology, Instituto Nacional de Neurología y Neurocirugía Manuel Velasco Suárez, Mexico City 14269, Mexico; maldonado.perla@quimica.unam.mx (P.D.M.); csilva@innn.edu.mx (C.A.S.-I.)

**Keywords:** neuroblastoma, garlic, S-allylcysteine, organosulfur compounds

## Abstract

Garlic (*Allium sativum*) has been used in alternative medicine to treat several diseases, such as cardiovascular and neurodegenerative diseases, cancer, and hepatic diseases. Several publications have highlighted other features of garlic, including its antibacterial, antioxidative, antihypertensive, and antithrombotic properties. The properties of garlic result from the combination of natural compounds that act synergistically and cause different effects. Some garlic-derived compounds have been studied for the treatment of several types of cancer; however, reports on the effects of garlic on neuroblastoma are scarce. Neuroblastoma is a prevalent childhood tumor for which the search for therapeutic alternatives to improve treatment without affecting the patients’ quality of life continues. Garlic-derived compounds hold potential for the treatment of this type of cancer. A review of articles published to date on some garlic compounds and their effect on neuroblastoma was undertaken to comprehend the possible therapeutic role of these compounds. This review aimed to analyze the impact of some garlic compounds on cells derived from neuroblastoma.

## 1. Neuroblastoma

Neuroblastoma (NB) is a tumor derived from neural crest tissue. NB develops from sympathetic nervous system cells, specifically from sympathoadrenal progenitor cells [1]. Tumors appear mainly on the suprarenal medulla; signs and clinical symptoms can range from benign tumors to severe cases due to tumor metastasis. Over 650 patients are diagnosed in the United States every year, with a prevalence of 1 case per 7000 live births and an incidence of approximately 10.54 cases per million per year in children under 15 years of age [2,3]. NB prognosis is based on the extent of tumor differentiation, the presence or absence of stroma, the age of the patient, and the status of the MYCN oncogene and chromosome 11q.21,22. The patients are classified into four groups: (I) very low risk, (II) low risk, (III) intermediate risk, and (IV) high risk [4,5]. In 90% of cases, low-risk patients have a favorable prognosis, whereas 60% of the high-risk patients have an unfavorable prognosis [6]. Moreover, treatment responses range from total remission to multiple drug resistance and severe toxicity [7,8]. It has been reported that half of patients with high-risk NB do not respond to first-line treatment or face relapse within the 2 years following the treatment [9,10,11].

NB is a heterogeneous disease from biological, clinical, morphological, and genetic perspectives, which poses a challenge for developing a universal treatment [12].

In this context, it is imperative to identify new therapies for childhood cancer that are affordable for the entire population and do not affect the patient’s quality of life. Garlic compounds have therapeutic uses, and their study raises the possibility of their use as adjuvants in traditional NB treatment. Table 1 summarizes the studies that describe the effect of garlic-derived compounds on NB. Although there are studies on the antitumor effect of some garlic compounds on different types of cancer, more studies are required to describe the molecular mechanism involved in the antitumor effect on NB.

## 2. Garlic

Garlic (*Allium sativum*) is a bulb belonging to the family Liliaceae, which comprises approximately 600 species. Although garlic originated in Asia, it is now distributed worldwide. Anatomically, garlic consists of a bulb, commonly known as the head of garlic, which is divided into small bulbs known as garlic cloves [24].

Garlic has been used therapeutically since ancient times. Egyptian crypts contain the oldest visible inscriptions regarding the existence of garlic. The Ebers papyrus mentioned that garlic was used to treat over 30 diseases [25,26]. According to numerous studies, garlic is an effective drug in the prevention and treatment of several diseases, such as atherosclerosis, owing to its lipid-lowering effect, moderate decrease in arterial pressure, and fibrinolytic and platelet anti-aggregation activities. Garlic also possesses antioxidant, hypotensive, antimicrobial, antifungal, antitumorigenic, and immunomodulatory properties [27,28,29,30,31]. Among the numerous compounds in garlic, organosulfur compounds are of particular importance. In intact bulbs, the main component is alliin, but compounds such as S-glutathione, γ-glutamyl-S-allyl-cysteine, and γ-glutamyl-S-allylmercapto-L-cysteine can also be found. When garlic is cut, crushed, or ground, the enzyme alliinase catalyzes the formation of allicin from alliin. Approximately 1 mg of alliin corresponds to 0.45 mg of allicin. In addition to organosulfur compounds, garlic bulbs contain mineral salts (selenium, phosphorus, copper, and potassium), sugars, lipids, essential amino acids, saponins, terpenes, vitamins, enzymes, flavonoids, and other phenolic compounds [32].

An antioxidant activity has been reported for all garlic compounds, with S-allylcysteine (SAC) and allicin showing the highest activity. These compounds effectively inhibit free radical formation, enhance the endogenous radical scavenging mechanism, and increase the cellular antioxidant enzymes [33]. The antioxidant properties of garlic and its compounds are of great interest in relation to their anti-atherogenic, anti-hepatotoxic, and anti-cancer effects. However, the effects of garlic compounds on human health may occur in addition to their antioxidant properties. Garlic and its different commercial preparations are the most widely studied herbal supplements. Furthermore, the use of garlic constitutes the second most common complementary therapy, as it has been employed to reduce hypercholesterolemia and hypertension. In addition, hepatoprotective and neuroprotective properties have been attributed to garlic [34,35].

## 3. Types of Garlic Preparations

Garlic-based products are popular among the various dietary supplements available in the market. Garlic and its compounds are sold in several presentations [36].

Garlic oil: This presentation is rich in diallyl disulfide (DADS) and diallyl trisulfide (DATS). [37]. Garlic oil maceration: This presentation is rich in compounds such as vinyldithiin, ajoene, and other organosulfur compounds; it also contains residual amounts of alliin [38]. Garlic powder: The main organosulfur compound in raw and powdered garlic is alliin [39,40]. Aged garlic extract (AGE): This presentation has an increase in the amount of SAC, which becomes the main AGE compound. SAC is a stable water-soluble compound with a high bioavailability and high antioxidant properties [41]. 

**Figure 1:**
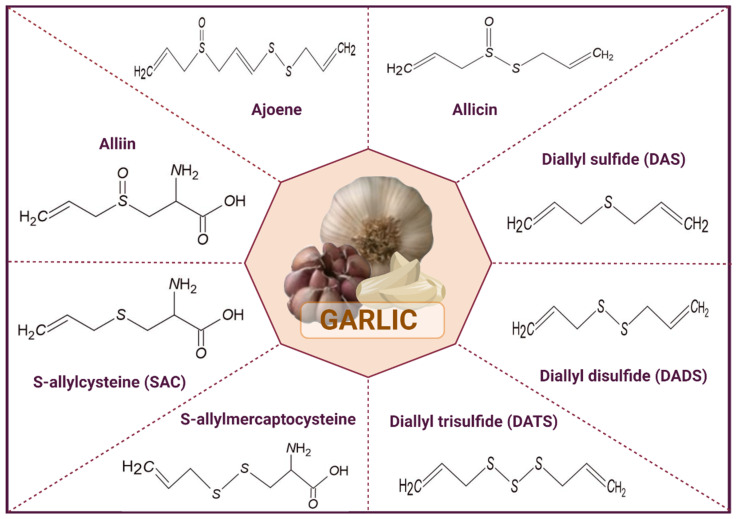
Structures of some of the organosulfur compounds present in garlic.

The beneficial effects of garlic are attributed to the presence of organosulfur compounds (Figure 1), which represent approximately 1% dry weight; among these compounds, alliin is the most abundant [42,43]. Several epidemiological studies have suggested that eating garlic reduces the risk of certain types of cancer, including gastrointestinal cancer [44,45]. Studies on animals have provided evidence that garlic and its compounds inhibit carcinogenesis through some events, including the induction of apoptosis, inhibition of cell proliferation, scavenging of reactive oxygen species (ROS), increasing the activity of enzymes such as glutathione-S-transferase, and reducing the tumor size [46]. The effects of garlic-derived compounds on different types of cancer have been investigated; however, studies on their antitumor effects on NB are scarce. 

## 4. Antioxidant Activity of Garlic and Garlic Compounds 

It has been demonstrated that natural compounds such as garlic and garlic-derived compounds can upregulate detoxifying enzymes and the enzymes involved in the antioxidant activity, thus decreasing the intracellular ROS [46,47]. The two allyl derivatives of garlic DAS and DADS can modulate the phase II detoxifying enzymes. Thus, it was demonstrated that DADS in high doses increased the activity of quinone reductase (QR) and glutathione S-transferase (GST) in the gastrointestinal tract of rats; therefore, the dose used in this study was close to that achieved through the human consumption of garlic [48]. The effects of garlic oil, DADS, and DAS were demonstrated in rats fed with a high-fat diet; thus, DAS and DADS increased GST activity, whereas DADS only increased 7-pent-oxyresorufin O-dealkylase activity. In addition, DADS and DAS increased the hepatic glutathione reductase (GR) activity; garlic oil, DADS, and DAS increased red blood cell GSH concentration; and, finally, garlic oil improved the superoxide dismutase (SOD) activity [49]. In the induced oral tumour tissue from hamsters treated with aqueous garlic extract, a decrease in lipid peroxidation, followed by a significant increase of reduced glutathione (GSH), glutathione peroxidase (GPx), and GST [50] was observed. AGE exhibited the suppression of H_2_O_2_ and O_2_^−^ generation in endothelial cells and significantly increased the activities of SOD, catalase, and GPx [51]. In rats with adriamycin-induced cardiotoxicity, the fresh garlic homogenate treatment demonstrated a significant decrease of lipid peroxidation and a significant increase in myocardial catalase, SOD, and GPx [52]. The treatment with allicin to cultured endothelial cells up-regulated phase II detoxifying enzymes, such as heme oxygenase-1 (HO-1), thioredoxin reductase 1 and 2, and the glutamate-cysteine ligase modifier subunit, which is a limiting enzyme in glutathione biosynthesis, preventing ROS damage [53]. Treatment with AGE significantly increased the antioxidant defence systems, as indicated by increased levels of antioxidant enzymes catalase and SOD and decreased lipid peroxidation in liver and kidney tissues [54]. Alliin reduced the receptor activator of nuclear factor-κB ligand (RANKL)-induced osteoclastogenesis and decreased the degeneration of ROS and NADPH oxidase 1 [55]. These data suggest that garlic and garlic-derived compounds could be effective as antioxidants and play an important role against oxidative stress.

## 5. Allicin and NB

Allicin is a sulfur compound produced upon garlic tissue damage by alliin in a reaction catalyzed by the enzyme alliinase [56]. The effects of this compound on some cancers such as osteosarcoma, melanoma, glioblastoma, colon cancer, and liver cancer have been studied [57,58,59,60,61,62,63]. However, there are few reports on its use and mechanism of action on NB.

Allicin has been shown to decrease cell viability and the proliferation of human NB cell lines. After 24 h of exposure to allicin, the induction of poly (ADP-ribose) polymerase (PARP) was observed; this enzyme, among other functions, participates in programmed cell death. Another crtitical effect reported is the inhibition of ornithine decarboxylase, an enzyme that has oncogenic abilities [64], as it causes an increase in polyamines that are linked to the hyperproliferation of neoplastic cells [65]. This inhibiting effect has been reported for the SK-NFI, SK-N-AS, SK-N-Be, and Kelly cell lines treated with allicin concentrations of 12.5 and 25 μM [13].

Treatment of the SK-N-SH cell line with 5 μmol/l allicin for 24 h induced apoptosis by activating the p38 MAPK pathway and the subsequent release of cytochrome c (cyt c) [14].

**Figure 2:**
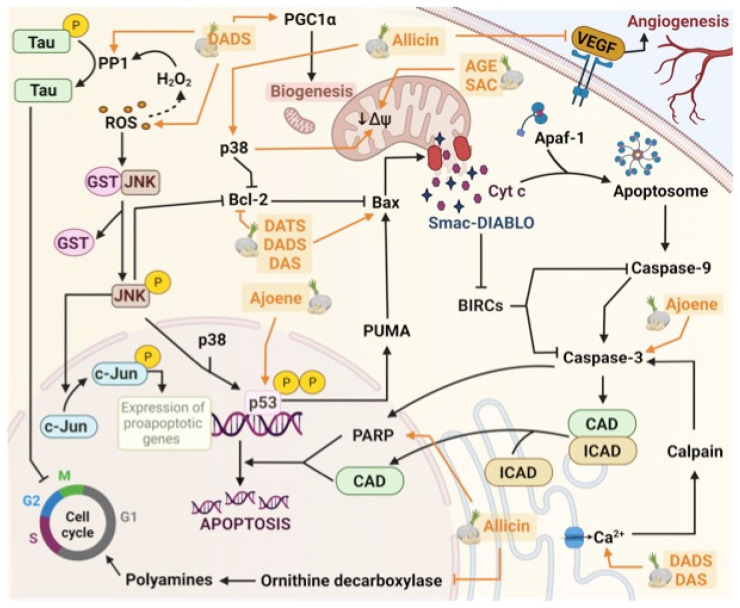
Schematic diagram of the molecular mechanisms of the effects of organosulfur compounds of garlic on the neuroblastoma.

Another report on the antitumor properties of allicin demonstrated that the combined therapy with allicin (10 mg/kg/day) and cyclophosphamide decreased the size of induced tumors in BALB/c-nu/nu mice bearing an SH-SY5Y NB cell line. Following combined therapy, tumor cell proliferation decreased (as assessed by histology) and the survival of the mice bearing the tumors increased. Moreover, the combined therapy (allicin+cyclophosphamide) increased the number of CD4+, CD8+, and NK cells, as well as the IFN-γ levels, in the serum. Interestingly, both the mRNA and protein levels of vascular endothelial growth factor (VEGF) decreased [15]. VEGF is a key molecule in the angiogenesis process and has been considered as a therapeutic target in NB [66]. These findings suggest that natural compounds derived from garlic may exhibit a synergistic effect when combined with conventional chemotherapeutic drugs (Figure 2).

## 6. Ajoene and NB

Ajoene is a compound derived from allicin, which exists as cis(Z)-ajoene and trans(E)-ajoene isomers. The antitumor properties of ajoene have been reported both in vitro and in vivo, but its effects on NB have not been explored much [67]. Only one report has shown the antitumor effect of Z-ajoene on the SK-N-AS cell line through induction of apoptosis, which was evinced by the increase in annexin V, propidium iodide, active caspase-3, and p53 6 h after the treatment [16]. This report is interesting as it suggests a p53-dependent mechanism of action (Figure 2).

## 7. SAC and NB

As previously mentioned, AGE is one of the most popular presentations of garlic, with SAC being a major component.

Welch et al. used the LA-N-5 human NB cell line to evaluate the antitumor effect of SAC. The cells were treated with SAC at 200, 400, 800, and 1600 μg/mL for 8 days. Decreased cell proliferation was observed from 400 μg/mL of SAC, thus demonstrating the antiproliferative potential of this compound. However, based on the evaluated morphological, biochemical, and molecular markers, the treatment failed to differentiate the NB cells [17].

Another study used the SJ-NK-P and IMR5 cell lines, and showed that SAC affects the mitochondrial membrane potential (Figure 2). The cells were treated with 1 and 2 mg/mL AGE, and the membrane potential (ΔΨm) and chemical gradient (ΔpHm), expressed in mV, were evaluated. AGE induced a decrease in ΔΨm of up to 120 mV, and the 58 ΔpHm value decreased by 10 mV. Changes in these parameters were accompanied by increased glutathione oxidation, as evinced by the decreased amount of reduced glutathione. After treatment of the cells with 20 mM SAC for 48 h, the number of apoptotic cells increased and the cell cycle was arrested at the G1 phase [18].

To date, few studies have been conducted on the use of SAC in cancer models. The available data support the potential use of AGE and SAC in the treatment of this disease.

## 8. DAS, DADS, DATS, and NB

The effects of DAS, DADS, and DATS on NB have been reported; however, the mechanism of action remains unclear.

Filomeni et al. demonstrated that DADS (50 µM for 24 h) induced apoptosis through the downregulation of the mitochondrial pathway (Bcl-2, release of cyt c into the cytosol, and activation of caspase-9) in association with the activation of the JNK/c-Jun pathway in SH-SY5Y cells (Figure 2) [19].

Karkamar et al. observed an increase in the intracellular Ca^2+^ levels, which triggered cell death due to endoplasmic reticulum stress in SH-SY5Y cells treated with DAS and DADS. The increase in intracellular Ca^2+^ upregulated the expression of the Ca^2+^-dependent protease calpain in the cells treated with DAS and DADS (Figure 2). The overexpression of calpain is associated with changes in the Bax/Bcl-2 ratio, which causes cell death through the release of cyt c from the mitochondria. The Bax/Bcl-2 ratio increased in the SH-SY5Y cells after treatment with DAS and DADS [20]. 

Aquilano et al. treated SH-SY5Y cells with 50 µmol/L DADS and observed that DADS induced the disruption of the cytoskeleton (Figure 2). The cctin levels decreased significantly 6 h after the treatment, and significant disruption of the microtubule network along with actin loss was observed by immunofluorescence in DADS-treated cells. These changes in the cytoskeleton could be explained by Tau protein dephosphorylation mediated by the protein phosphatase 1 (PP1) in a ROS-dependent pathway after treatment with DADS [21].

Pagliei et al. described the effects of the peroxisome proliferator-activated receptor-gamma co-activator 1 alpha (PGC1α) as an anti-apoptotic agent in DADS-treated SH-SY5Y cells (50 µM for 3, 6, and 9 h). DADS promoted the activation of PGC1α in a time-dependent manner, reaching a maximum expression 9 h after treatment. The activation of the PGC1α protein favored mitochondrial biogenesis (increase in mitochondrial mass) and reduced the apoptotic effects of DADS. PGC1α has been linked to both pro- and anti-cancer effects. In prostate, skin, breast, ovarian, liver, and colon cancers, it has been shown to promote mitochondrial biogenesis, thus increasing the size and proliferation of the tumors. These effects are associated with ROS levels and other oxidative stress indicators, which either activate or inhibite PGC1α and protect the mitochondria from malfunctioning due to oxidative stress. Given the anti-apoptotic response in the treated NB cells, the expression of PGC1α as an anti- or pro-apoptotic agent would depend on the type of cancer. Specifically, in the NB cells, PGC1α showed an anti-apoptotic response and decreased the efficacy of treatment with DADS (and possibly with other drugs that promote the death of cancer cells through apoptosis) [22]. 

Jurkowska et al. treated glioblastoma (U87MG) and NB (SH-SY5Y) cells with DATS. In U87MG cells, exposure to 100 µM DATS caused a decrease in Bcl-2 and increased hydrogen sulfide (H_2_S) production, which was associated with increased levels of GSH. The production of H_2_S could be associated with the increased activity of proteins that promote the transfer of sulfur atoms (MPST and rhodanese), forming sulfane sulfur; the latter would be transferred to the cysteine residues of Bcl-2. In NB cells, the Bcl-2 inactivation mechanism could not be associated with the apoptotic effect found in the study due to the low cysteine levels in these cells. However, ROS production could also support this mechanism, which would cause Bcl-2 inactivation through phosphorylation. The most significant antiproliferative activity was observed at 48 h [23]. 

Treatment with some garlic compounds can induce apoptosis in tumor cells. Ajoene increases p53 expression, which is phosphorylated (P) and activates the p53 upregulated modulator of apoptosis (PUMA). In the cytoplasm, PUMA activates pro-apoptotic proteins, such as B-cell lymphoma 2 (Bcl-2)-like protein 4 (Bax), which create a pore in the outer mitochondrial membrane and trigger the release of cyt c and the second mitochondria-derived activator of caspase/direct inhibitor of apoptosis protein (IAP)-binding protein with a low pI (Smac-DIABLO). DAS, DADS, and DATS also increase Bax and decrease Bcl-2, which enhance the effect. p38 activated by allicin also inhibits Bcl-2, and like SAC and AGE, it decreases the mitochondrial membrane potential. Cyt c in the cytosol induces the oligomerization of apoptotic peptidase activating factor 1 (Apaf-1). Apaf-1, cyt c, and adenosine triphosphate (ATP) form the apoptosome, which activates caspase-9. Activated caspase-9 leads to the processing of effector caspases, such as caspase-3. Caspase-3 can also be activated by calpain, which is first activated by the increase in free intracellular Ca^2+^ mediated by DAS and DADS. Smac-DIABLO inhibits survival signals through the downregulation of baculoviral inhibitors of apoptosis repeat containing (BIRC) proteins and allows for the activation of caspase-3. Activated caspase-3 degrades the inhibitor of caspase-activated DNase (ICAD). Free CAD cleaves the DNA into internucleosomal fragments. Caspase-3 also causes the cleavage of PARP, leading to DNA fragmentation and inhibition of the repair mechanisms. Allicin increases PARP levels. Apoptosis could also be induced through the c-Jun NH_2_-N-terminal kinase (JNK/c-Jun) pathway. DADS could increase ROS levels; under stress conditions, GST and JNK disassociate, which leads to JNK activation by phosphorylation. Activated JNK leads to c-Jun activation by phosphorylation and the expression of pro-apoptotic genes. Moreover, the increase in ROS, such as hydrogen peroxide (H_2_O_2_), could cause Tau dephosphorylation catalyzed by protein phosphatase 1 (PP1). Tau dephosphorylation contributes to cell cycle arrest. The cell cycle can also be inhibited by allicin, which reduces ornithine decarboxylase levels. Allicin also reduces vascular endothelial growth factor (VEGF) levels, which leads to decreased angiogenesis. Finally, DADS also increases the levels of the transcriptional co-activator peroxisome proliferator activated receptor gamma co-activator 1 alpha (PGC1α), a factor that promotes mitochondrial biogenesis. PGC1α has been linked to both pro- and anti-cancer effects, and the expression of PGC1α as an anti- or pro-apoptotic agent would depend on the type of cancer. 

## 9. Effects of Garlic on Neurodegenerative Diseases

Several neurodegenerative diseases such as Alzheimer’s, Parkinson’s, and Huntington’s diseases and amyotrophic lateral sclerosis present hallmarks of degenerative processes (e.g., oxidative stress, mitochondrial damage, inflammation, and apoptosis), and treatment with several natural compounds is expected to delay the onset and reduce the symptoms. Thus, in the Alzheimer’s mouse model (Tg2576) that overexpressed a mutant form of the amyloid precursor protein, the AGE decreased cerebral plaques and detergent-resistant and soluble β-amyloid peptide (Aβ)-species, reduced neuroinflammation, and reduced phosphorylation-induced conformational change in tau protein [68]. AGE ameliorated Aβ-induced neurotoxicity in PC12 cells by preventing intracellular ROS accumulation. Moreover, AGE also improved learning and memory deficits in ICV Aβ injected mice [69]. Treatment with AGE in rats injected with Aβ significantly improved the working memory and significantly ameliorated the loss of cholinergic neurons, and increased the vesicular glutamate transporter 1 and glutamate decarboxylase levels in the hippocampus [70]. Pre-treatment of AGE ameliorated the cognitive dysfunction in Aβ-induced neurotoxicity rats and increased the activities of SOD and GPx, and reduced the lipid peroxidation levels in rat brain homogenates [71]. 1-Methyl-4-phenylpyridinium (MPP^+^) caused nigrostriatal dopaminergic neurotoxicity, and it is widely used as a model of Parkinson’s disease. Thus, SAC protected against the oxidative stress induced by MPP^+^ in the mouse striatum, by improved hyperlocomotion, decreased lipid peroxidation and ROS production, and increased SOD1 activity. [72]. Garlic extract protected the dopaminergic neurons in 6-hydroxydopamine (6-OHDA) injected rats (which is used as a model for Parkinson’s disease) by improving 6-OHDA-induced motor dysfunctions and alleviating non-motor deficits, including memory impairment and anxiety in rats [73]. In rat brain synaptosomes, SAC, which is a compound in AGE, decreased lipid peroxidation and mitochondrial dysfunction induced by 3-nitropropionic acid, which produces oxidative and nitrosative stress and evokes an experimental model of Huntington’s disease. [74]. Moreover, SAC was used in an animal model of Huntington’s disease, and SAC treatment increased SOD1 and 2, and decreased lipid peroxidation and mitochondrial dysfunction and prevented behavioural alterations [75]. The effects of DATS, a major constituent in garlic oil, were evaluated in a transgenic mouse model of amyotrophic lateral sclerosis. DATS treatment in SOD1-G93A transgenic mice induced HO-1 and reduced glial fibrillary acidic protein (GFAP) expression in the lumbar spinal cord [76]. This data suggest the possible roles of garlic and garlic-derived compounds as antioxidant agents, and it is also suggested that they can act as a neuroprotector in several neurodegenerative diseases. Nevertheless, further studies are warranted to verify the effects, dosages, and efficacy of garlic, either alone or in combination with other garlic-derived compounds, as a possible treatment alternative for neurodegenerative diseases.

## 10. Conclusions

Garlic has been shown to have therapeutic properties in the treatment of several neurodegenerative diseases, including cancer. The molecular mechanisms through which garlic exerts its antioxidative and antitumor effects have not been fully described and depend not only on the type of compound, but also on the type of cancer to be treated. NB is a childhood tumor of public health importance; therefore, it is imperative to find new therapeutic alternatives that are affordable and do not affect the patients’ quality of life. Although reports are scarce, garlic-derived compounds have shown antioxidative and antitumor effects on NB-derived cells, which make them good candidates to be used as adjuvants in treatment. However, further studies using animal models and patient trials are needed.

Given the lack of information to date, the exploration of garlic-derived compounds as adjuvants in the treatment of NB opens the door to future studies that could help clarify the molecular mechanisms of action. 

## Figures and Tables

**Table 1:** Effect of aged garlic extract (AGE) and garlic compounds on neuroblastoma.

Compound Used	Experimental Model	Main Findings	Reference
Allicin	SK-NFI, SK-N-AS, SK-N-Be, and Kelly cell lines	Induction of programmed cell death by the increase of poly (ADP-ribose) polymerase (PARP).	[13]
Allicin	SK-N-SH cell line	Induction of apoptosis by activating the p38 MAPK pathway and the release of cytochrome c.	[14]
Allicin	BALB/c-nu/nu mice	Tumour cell proliferation decreased; increase the number of CD4+, CD8+, and NK cells and IFN-γ levels in the serum; and decrease in mRNA and protein levels of VEGF.	[15]
Z-ajoene	SK-N-AS cell line	Increase active caspase-3 and p53.	[16]
SAC	LA-N-5 human NB cell line	Decrease cell proliferation.	[17]
SAC	SJ-NK-P and IMR5 cell lines	Induction of apoptosis and cell cycle arrest in the G1 phase.	[18]
AGE	SJ-NK-P and IMR5 cell lines	Decrease the mitochondrial membrane potential and increased glutathione oxidation.	[18]
DADS	SH-SY5Y cell line	Release of cytochrome c, and activation of caspase-9 in association with the activation of the JNK/c-Jun pathway.	[19]
DAS and DADS	SH-SY5Y cell line	Both compounds increase the intracellular Ca^2+^ and induce cell death through the release of cytochrome c.	[20]
DADS	SH-SY5Y cell line	Disruption of the cytoskeleton by Tau protein dephosphorylation.	[21]
DADS	SH-SY5Y cell line	Activation of PGC1α.	[22]
DATS	U87MG and SH-SY5Y cell line	Decrease of Bcl-2, and increased hydrogen sulfide production and ROS production.	[23]

AGE: Aged garlic extract; DADS: diallyl disulphide; DAS: diallyl sulphide; DATS: diallyl trisulfide; IFN-γ: Interferon-γ; JNK/c-Jun: c-Jun NH2-N-terminal kinase; NK: Natural killers; PGC1α: peroxisome proliferator-activated receptor-Gamma co-activator 1 alpha; ROS: reactive oxygen species; SAC: S-allylcysteine; VEGF: vascular endothelial growth factor.
